# Supplementary material for: Modeling glioblastoma heterogeneity as a dynamic network of cell states
Source: Mol Syst Biol. 2021 Sep 16;17(9):e10105. doi: 10.15252/msb.202010105 (PMC8444284; doi:10.15252/msb.202010105)
Supplement: Supplementary file 6 — Source Data for Figure 5 [file MSB-17-e10105-s004.zip › Figure5A_sourcedata/GSEA_3071/hallmarks_stateIII.GseaPreranked.1623742540963/gsea_report_for_na_neg_1623742540963.html]

Report for na\_neg 1623742540963 [GSEA]

| GS  follow link to MSigDB | GS DETAILS | SIZE | ES | NES | NOM p-val | FDR q-val | FWER p-val | RANK AT MAX | LEADING EDGE || 1 | HALLMARK\_EPITHELIAL\_MESENCHYMAL\_TRANSITION | Details ... | 114 | -0.56 | -3.53 | 0.000 | 0.000 | 0.000 | 457 | tags=47%, list=12%, signal=52% |
| 2 | HALLMARK\_COAGULATION | Details ... | 36 | -0.57 | -2.74 | 0.000 | 0.000 | 0.000 | 717 | tags=61%, list=18%, signal=74% |
| 3 | HALLMARK\_MYOGENESIS | Details ... | 57 | -0.49 | -2.73 | 0.000 | 0.000 | 0.000 | 359 | tags=37%, list=9%, signal=40% |
| 4 | HALLMARK\_ANGIOGENESIS | Details ... | 19 | -0.65 | -2.54 | 0.000 | 0.000 | 0.000 | 507 | tags=58%, list=13%, signal=66% |
| 5 | HALLMARK\_KRAS\_SIGNALING\_UP | Details ... | 56 | -0.45 | -2.48 | 0.000 | 0.000 | 0.001 | 933 | tags=54%, list=24%, signal=69% |
| 6 | HALLMARK\_UV\_RESPONSE\_DN | Details ... | 50 | -0.43 | -2.27 | 0.000 | 0.001 | 0.008 | 1251 | tags=60%, list=32%, signal=87% |
| 7 | HALLMARK\_HYPOXIA | Details ... | 76 | -0.38 | -2.24 | 0.000 | 0.001 | 0.009 | 998 | tags=50%, list=25%, signal=66% |
| 8 | HALLMARK\_IL2\_STAT5\_SIGNALING | Details ... | 63 | -0.38 | -2.11 | 0.000 | 0.003 | 0.028 | 1078 | tags=51%, list=27%, signal=69% |
| 9 | HALLMARK\_ESTROGEN\_RESPONSE\_EARLY | Details ... | 62 | -0.38 | -2.08 | 0.000 | 0.004 | 0.035 | 1080 | tags=55%, list=27%, signal=74% |
| 10 | HALLMARK\_INFLAMMATORY\_RESPONSE | Details ... | 45 | -0.40 | -2.03 | 0.001 | 0.006 | 0.056 | 510 | tags=38%, list=13%, signal=43% |
| 11 | HALLMARK\_KRAS\_SIGNALING\_DN | Details ... | 32 | -0.43 | -2.01 | 0.001 | 0.006 | 0.064 | 1342 | tags=63%, list=34%, signal=94% |
| 12 | HALLMARK\_ESTROGEN\_RESPONSE\_LATE | Details ... | 51 | -0.35 | -1.91 | 0.003 | 0.011 | 0.124 | 985 | tags=47%, list=25%, signal=62% |
| 13 | HALLMARK\_APOPTOSIS | Details ... | 56 | -0.33 | -1.80 | 0.002 | 0.025 | 0.282 | 686 | tags=36%, list=17%, signal=43% |
| 14 | HALLMARK\_TNFA\_SIGNALING\_VIA\_NFKB | Details ... | 69 | -0.31 | -1.80 | 0.005 | 0.024 | 0.290 | 1053 | tags=43%, list=27%, signal=58% |
| 15 | HALLMARK\_APICAL\_JUNCTION | Details ... | 51 | -0.32 | -1.69 | 0.020 | 0.052 | 0.547 | 743 | tags=37%, list=19%, signal=45% |
| 16 | HALLMARK\_GLYCOLYSIS | Details ... | 57 | -0.28 | -1.57 | 0.034 | 0.099 | 0.808 | 888 | tags=37%, list=23%, signal=47% |
| 17 | HALLMARK\_BILE\_ACID\_METABOLISM | Details ... | 19 | -0.36 | -1.44 | 0.087 | 0.199 | 0.980 | 1386 | tags=63%, list=35%, signal=97% |
| 18 | HALLMARK\_COMPLEMENT | Details ... | 51 | -0.26 | -1.41 | 0.102 | 0.219 | 0.988 | 1528 | tags=53%, list=39%, signal=85% |
| 19 | HALLMARK\_ADIPOGENESIS | Details ... | 56 | -0.23 | -1.28 | 0.208 | 0.366 | 0.999 | 770 | tags=29%, list=20%, signal=35% |
| 20 | HALLMARK\_APICAL\_SURFACE | Details ... | 16 | -0.34 | -1.27 | 0.193 | 0.361 | 0.999 | 860 | tags=44%, list=22%, signal=56% |
| 21 | HALLMARK\_FATTY\_ACID\_METABOLISM |  | 29 | -0.28 | -1.25 | 0.224 | 0.369 | 1.000 | 710 | tags=31%, list=18%, signal=38% |
| 22 | HALLMARK\_MITOTIC\_SPINDLE |  | 50 | -0.23 | -1.23 | 0.229 | 0.379 | 1.000 | 1718 | tags=56%, list=44%, signal=98% |
| 23 | HALLMARK\_CHOLESTEROL\_HOMEOSTASIS |  | 23 | -0.27 | -1.13 | 0.330 | 0.526 | 1.000 | 1277 | tags=48%, list=32%, signal=70% |
| 24 | HALLMARK\_TGF\_BETA\_SIGNALING |  | 18 | -0.29 | -1.12 | 0.349 | 0.520 | 1.000 | 1284 | tags=50%, list=33%, signal=74% |
| 25 | HALLMARK\_PROTEIN\_SECRETION |  | 21 | -0.26 | -1.07 | 0.374 | 0.581 | 1.000 | 1188 | tags=43%, list=30%, signal=61% |
| 26 | HALLMARK\_P53\_PATHWAY |  | 69 | -0.19 | -1.07 | 0.392 | 0.565 | 1.000 | 1907 | tags=59%, list=49%, signal=113% |
| 27 | HALLMARK\_INTERFERON\_GAMMA\_RESPONSE |  | 39 | -0.19 | -0.97 | 0.520 | 0.703 | 1.000 | 1103 | tags=36%, list=28%, signal=49% |
| 28 | HALLMARK\_G2M\_CHECKPOINT |  | 55 | -0.17 | -0.94 | 0.556 | 0.718 | 1.000 | 325 | tags=15%, list=8%, signal=16% |
| 29 | HALLMARK\_MTORC1\_SIGNALING |  | 64 | -0.16 | -0.89 | 0.618 | 0.770 | 1.000 | 373 | tags=14%, list=9%, signal=15% |
| 30 | HALLMARK\_XENOBIOTIC\_METABOLISM |  | 38 | -0.18 | -0.88 | 0.625 | 0.762 | 1.000 | 717 | tags=24%, list=18%, signal=29% |
| 31 | HALLMARK\_ANDROGEN\_RESPONSE |  | 28 | -0.19 | -0.87 | 0.609 | 0.746 | 1.000 | 1393 | tags=43%, list=35%, signal=66% |
| 32 | HALLMARK\_PI3K\_AKT\_MTOR\_SIGNALING |  | 23 | -0.20 | -0.83 | 0.654 | 0.784 | 1.000 | 174 | tags=13%, list=4%, signal=14% |
| 33 | HALLMARK\_INTERFERON\_ALPHA\_RESPONSE |  | 23 | -0.20 | -0.83 | 0.678 | 0.765 | 1.000 | 1779 | tags=57%, list=45%, signal=103% |
| 34 | HALLMARK\_PEROXISOME |  | 21 | -0.18 | -0.74 | 0.755 | 0.845 | 1.000 | 1741 | tags=52%, list=44%, signal=94% |
| 35 | HALLMARK\_HEME\_METABOLISM |  | 45 | -0.12 | -0.62 | 0.902 | 0.946 | 1.000 | 1015 | tags=27%, list=26%, signal=36% |
| 36 | HALLMARK\_UV\_RESPONSE\_UP |  | 39 | -0.10 | -0.49 | 0.965 | 0.995 | 1.000 | 310 | tags=8%, list=8%, signal=8% |
| 37 | HALLMARK\_E2F\_TARGETS |  | 49 | -0.07 | -0.38 | 0.996 | 0.997 | 1.000 | 2437 | tags=63%, list=62%, signal=164% |
Table: Gene sets enriched in phenotype **na **[plain text format]****
